# Characterization of Starches From *Pueraria lobata* and *Pueraria thomsonii*: Structural and Physicochemical Properties and Comparison With Commercial Starches

**DOI:** 10.1155/ijfo/1032983

**Published:** 2025-12-15

**Authors:** Jianbin Shi, Haofeng Zou, Yong Sui, Tian Xiong, Xueling Chen, Chuanhui Fan, Xin Mei

**Affiliations:** ^1^ Key Laboratory of Agricultural Products Cold Chain Logistics, Ministry of Agriculture and Rural Affairs, Institute of Agro-Products Processing and Nuclear Agricultural Technology, Hubei Academy of Agricultural Sciences, Wuhan, China, hbaas.com; ^2^ College of Chemical Engineering, Xiangtan University, Xiangtan, China, xtu.edu.cn

**Keywords:** digestive properties, physiochemical property, *Pueraria*, starches

## Abstract

**Background:**

The *Pueraria lobata* (Willd.) Ohwi and *Pueraria thomsonii* Benth. are widely distributed and considered medicinal and edible plants in China. To explore the potential use in the food and nonfood industry, the structural and physicochemical properties of *Pueraria* starches were studied, which included the composition, morphology and size distributions, crystal structure, freeze–thaw stability, and in vitro digestion.

**Results:**

The results indicated that *Pueraria* starches showed significantly higher (*p* < 0.05) amylose content and smaller average particle size *D*[3, 4] than commercial starches. The degree of crystallinity of KS, PBS1, and PBS2, characterized by C‐type diffraction patterns, was 28.27%, 24.97%, and 25.14%, respectively. PBS1 and PBS2 had higher paste temperatures than KS. The significantly higher (*p* < 0.05) water binding capacity was observed in PBS2 and KS at 99.87% and 98.64%, respectively. PBS1 demonstrated the highest oil binding capacity but lower freeze–thaw stability compared to KS and PBS2. KS exhibited a high RDS content (90.85%) and low SDS and RS contents (3.26% and 5.89%, respectively). PBS1 had the highest RS content (11.26%).

**Conclusions:**

This research establishes a theoretical foundation for developing *Pueraria* starch resources in both food and nonfood industry applications.

## 1. Introduction

*Pueraria*, a perennial leguminous vine, is predominantly distributed in subtropical and temperate regions [1–3]. *Pueraria* is extensively cultivated and receiving great attention for its beneficial, nutritional, and medical applications in Asia [4]. The species of *Pueraria lobata* (Willd.) Ohwi and *Pueraria thomsonii* Benth. are common in China and considered medicinal and edible plants. The cultivation area and production are estimated to exceed 700 thousand hectares and 200 thousand tons per year, respectively [2]. *Pueraria lobata* (Willd.) Ohwi, also known as kudzu, is rich in isoflavonoids, including puerarin, genistein, and formononetin, and has been widely used in traditional Chinese medicine to treat neck stiffness, lack of perspiration, and aversion to air drafts since ad 200 [5, 6]. Modern medicine has validated kudzu isoflavonoids′ biological activities, such as immunomodulatory activity, anti‐inflammatory activity, and anticancer properties [7–13]. Compared with kudzu, *Pueraria thomsonii* Benth. contains a high content of starch and is called edible kudzu or starch kudzu [14]. Both species are included in the Chinese Pharmacopeia (Committee for the Pharmacopeia of P.R. China, 2020), which stipulates puerarin content not less than 2.4% for *Pueraria lobata* (Willd.) Ohwi and 0.3% for *Pueraria thomsonii* Benth [15].

Modern research primarily focused on the active compounds in *Pueraria*. Large quantities of starch were discarded after the active compounds extraction, leading to resource wastage. Thus, utilizing starch is crucial for developing *Pueraria* resource. However, there are few industrial products on the market due to high prices and inherent limitations like high viscosity, hot paste instability, and retrogradation tendency [16]. Starch is one of the main components in *Pueraria*, ranging from 15% to 46% in the fresh root of *Pueraria* [17, 18]. The structural and physicochemical properties of starches determine their functionality in various applications [19]. Van Hung and Morita reported that kudzu starches (KSs) exhibited similar amylose content (22.2%–22.9%) but differ in other properties like *λ*_ma*x*_, blue value, degree of polymerization (DP), and chain number in amylose and amylopectin from Vietnam, Japan, and Korea. Additionally, the x‐ray diffraction (XRD) patterns of KS showed A‐type, C‐type, and B‐type, respectively. Xia et al. [20] reported that *Pueraria lobata* (Willd.) Ohwi starch has lower amylose content, clarity, crystallinity, peak temperature, gelatinization temperature range, and gelatinization enthalpy compared to *Pueraria thomsonii* Benth. starch. However, there is no distinct visual difference between KS and edible KS and no systematic research on the structural and physicochemical properties of them [21]. This makes it difficult to effectively control the quality of processed products with KS and edible KS as the main material. The *Pueraria* is usually harvested and consumed at different growth years. Extensive studies have showed the effect of growth years on the multiscale structure and practical features of starches. Growth years led to significant variations in amylose content, granule size, solubility, swelling power, gelatinization temperature, and digestibility of starches derived from sago, yam, and cassava [22]. The growth cycle of *Pueraria* (i.e., the duration of its growth years) is considerably longer than that of common starch sources, including sweet potato, corn, and cassava. As *Pueraria* tubers mature and grow, the fine structure and functional properties of their starch may undergo significant alterations. Considering the abovementioned, it is meaningful to study the structural and physicochemical properties of starches for their food industrial application. The aim of the present study was to investigate the structural and physicochemical properties of starches from *Pueraria lobata* (Willd.) Ohwi and two types of edible KS from *Pueraria thomsonii* Benth. harvested after 1 year and 3 years planting by scanning electron microscopy (SEM), XRD, freeze–thaw stability, and in vitro digestion.

## 2. Materials and Methods

### 2.1. Materials

KS from *Pueraria lobata* (Willd.) Ohwi and two types of edible KS from *Pueraria thomsonii* Benth. (harvested after 1 year and 3 years of planting, named PBS1 and PBS2, respectively) were provided by Hubei Zhongping Gegen Industry Development Co. LTD. (Xiangyang, China). The species confirmation of *Pueraria* was carried out by the technical worker of the company. Sweet potato starch (SPS), cassava starch (CF), and corn starch (CS) were purchased from a market in Wuhan. All the starch samples were kept in self‐sealing bags under room temperature with low humidity (< 60%). All chemicals and reagents in the present work are of analytical grade from the China National Pharmaceutical Group Corporation.

### 2.2. Proximate Composition of Starch

Proximate composition analysis included the determination of moisture, lipid, and ash contents, which were assayed using the official methods of AACC International: 44‐15A for moisture, 08‐01 for lipid, and 30‐10 for ash (AACC, 2000). Crude protein content was estimated from the total nitrogen content, which was measured using a protein analyzer according to the Kjeldahl method, with a conversion factor of 6.25 (*N* × 6.25). The total starch content was determined following AACC Approved Method 76‐13.01. Amylose content was quantified using the concanavalin A (Con A) precipitation method, with a Megazyme Amylose/Amylopectin Assay kit (Megazyme, Ireland).

### 2.3. Morphology and Size Distributions of Starch Granules

SEM (QUANTA 200, FEI, the Netherlands) was applied to characterize the morphology of the starch granule at an accelerating voltage of 20 kV. Starch samples were first sputter‐coated with gold and then observed using a SEM at a magnification of 2000× and a working distance of 10.1 mm. To ensure the reproducibility of the results, image acquisition was repeated three times under the same magnification settings.

The starch particle size distribution was analyzed using a laser particle size analyzer (Mastersizer 2000, Malvern, United Kingdom). Reverse osmosis water was used as the exclusive dilution medium in the tests. The 1.0 g starch sample was dispersed in 10 mL reverse osmosis water to form a primary starch suspension. Subsequently, this suspension was added dropwise into a sample cell containing about 350 mL reverse osmosis water until the obscuration value was above 10%. The refractive index of the dilution medium (RO water) was set to 1.33, while that of the starch was set to 1.52. Size distribution tests were carried out in triplicate.

### 2.4. XRD Measurement

The crystalline structures of starch samples were analyzed using a powder x‐ray diffractometer (X′Pert Pro MPD, Nalytical, the Netherlands). Diffractograms were obtained from 3° 2*θ* to 45° 2*θ* with a scanning speed of 8°/min and a scanning step of 0.02° [23]. The crystallinity and amorphous areas on the diffractograms were measured using Jade 6.0 (Materials Data, Inc., United States). The ratio of the crystallinity area to the total diffraction area was calculated as the degree of crystallinity (percentage).

### 2.5. Pasting Properties

The pasting properties of starch were determined using a rapid visco analyzer (RAV 4500, Perten, Australia). Three grams of starch were dispersed in 25 mL of distilled water in an RAV can, which is loaded onto the analyzer. The test procedure was set as follows: (1) stirring at 960 rpm for 10 s at 50°C and stirring at 160 rpm for 50 s at 50°C, (2) heating the temperature from 50°C to 95°C within 7.5 min at 160 rpm, (3) keeping at 95°C for 5 min at 160 rpm, (4) cooling the temperature from 95°C to 50°C within 7.5 min at 160 rpm, and (5) keeping at 50°C for 2 min at 160 rpm. The pasting parameters, including peak viscosity, trough viscosity, final viscosity, breakdown, and setback viscosities, were recorded.

### 2.6. Water and Oil Binding Capacity

Water and oil binding capacities were measured based on a reported method with modifications [24]. Five grams of starch (dry weight) were placed in a centrifugal tube with 50 g of water or soybean oil and agitated for 10 min at 25°C. After centrifuging at 1000 × g for 10 min, the precipitate was weighed. The water/oil binding capacity was calculated as follows:

wateroilbinding capacity %=m1−m0m0×100%,

where *m*_0_ is the weight of starch and *m*_1_ is the weight of precipitate after centrifuging.

### 2.7. Swelling Power and Solubility

Swelling power and solubility were measured according to a modified method [25]. Aqueous suspensions of starch (2% *w*/*v*) were stirred in a water bath at 60°C, 70°C, 80°C, and 90°C for 30 min separately. During heating, the samples were oscillated for 5 s every 2 min. After cooling to 25°C, the starch suspensions were centrifuged at 1000 × g for 20 min. The wet starch sediments were precisely weighed, and the supernatants were decanted and dried to a constant weight in an oven at 105°C. Swelling power and solubility were calculated as follows:

solubility%=m1m0×100%,swelling powerg/g=m2m0×1100−solubility/,

where *m*_0_ is the dry weight of starch in aqueous suspensions, *m*_1_ is the dry basis weight of starch in the supernatant, and *m*_2_ is the weight of wet starch sediments after centrifugation.

### 2.8. Freeze–Thaw Stability Measurement

Freeze–thaw stability was assessed by measuring syneresis of freeze–thawed starch gels using a previously described method with minor modifications [26]. A 5% starch solution was stirred in boiled water for 30 min. After cooling to room temperature, the starch paste was transferred into a centrifugal tube and frozen for 24 h at −18°C. After thawing at room temperature, the starch paste was centrifuged at 1500 × g for 10 min. The supernatant was discarded, and the sediment was weighed. This process was repeated five times. Syneresis was calculated as follows:

syneresis%=m1−m2m1×100%,

where *m*_1_ is the weight of the starch paste before centrifugation and *m*_2_ is the weight of the sediment after centrifugation.

### 2.9. In Vitro Digestibility of Starch

The in vitro digestibility was analyzed using a modified method of Englyst et al. [27, 28]. Briefly, starch samples (0.1 g) were mixed with 10 mL of 0.1 M sodium phosphate buffer (pH 6.8) in a centrifuge tube, which was gelatinized in boiling water for 30 min. After cooling to room temperature, the gelatinized starch was mixed with 5 mL of 0.01 M NaOH and 5 mL enzyme solution (containing 200 U/mL *α*‐amylase and 200 U/mL glucosidase, Shanghai Yuanye Biotechnology Co. Ltd., China) and incubated in a shaking water bath at 37°C. Aliquots (0.1 mL) were taken at 20 and 120 min. Each aliquot was then mixed with 0.9 mL of absolute ethanol to stop the reaction. Glucose content during hydrolysis was determined by the DNS method. The contents of rapidly digestible starch (RDS), slowly digestible starch (SDS), and resistant starch (RS) were calculated using the following equations.

RDS%=G20−FG×0.9TS×100%,SDS%=G120−G20×0.9TS×100%,RS%=100%−RDS−SDS,

where FG, *G*_20_, and *G*_120_ represent the free glucose in the starch sample and the glucose released within 20 min and 120 min, respectively, and TS represents the total starch in the sample.

### 2.10. Statistical Analysis

All tests were carried out in triplicate. Analysis of variance (ANOVA) was performed using Duncan′s multiple range test to compare treatment means at *p* < 0.05 using SPSS 26.0 (SPSS Inc., United States). Pearson′s correlation coefficients (*r*) were calculated for the various physicochemical properties by Origin 2021.

## 3. Results and Discussions

### 3.1. Composition of Starch

The main compositions of starch samples are shown in Table 1. For *Pueraria* starches, the purity of starch ranged from 87.44% to 98.24%, while the purity of starch in SPS, CF, and SC was 94.45%, 96.53%, and 87.24%, respectively. The moisture contents of all starch samples varied between 8.13% and 13.65%, which falls within the moisture level recommended for safe storage in the most producing countries. The variation in moisture content could be attributed to the extent of drying of the starches. The nonstarch constituents, including ash (0.23%–1.01%), protein (0.12%–0.36%), and lipids (1.05%–1.79%), were present in minor amounts; however, these components can still influence the physicochemical and functional properties of the starches. Significant differences in amylose content were observed among KS (22.92%), PBS1 (27.75%), and PBS2 (29.94%) (*p* < 0.05). Previous studies have reported similar amylose contents (20.6%–22.9%) for KSs from China, Vietnam, Japan, and Korea [29, 30]. Consistent with our findings, Xia et al. [20] noted that edible KS exhibits a higher amylose content compared to nonedible KS. Additionally, *Pueraria* starches had significantly higher amylose content (*p* < 0.05) than SPS (17.10%), CF (17.53%), and CS (18.96%). However, a separate study indicated that SPS has higher amylose content (34.4%) than *Pueraria* starches sourced from Hunan, Guangxi, and Jiangxi provinces in China [2]. The activities of enzymes involved in starch biosynthesis are susceptible to changes in environmental conditions and agronomic practices, and this variability is potentially a key driver of differences in amylose content [31]. Variations in amylose contents can occur because of different botanical sources. Amylose content plays an important role in the nutritional, functional, and quality characteristics of starch‐based products [14]. High‐amylose starches are typically characterized by strong gelling capacity and a greater tendency to undergo retrogradation [19, 32].

**Table 1:** Main composition of starches.

	Moisture (%)*	**Starch (%)**	**Ash (%)**	**Protein (%)**	**Lipid (%)**	**Amylose (%)**
KS	11.64 ± 0.09^c^	87.44 ± 2.43^c^	1.01 ± 0.03^a^	0.26 ± 0.06^b^	1.79 ± 0.21^a^	22.92 ± 0.57^c^
PBS1	8.13 ± 0.11^f^	98.24 ± 0.23^a^	0.30 ± 0.02^d^	0.12 ± 0.04^c^	1.23 ± 0.04^c^	27.75 ± 1.17^b^
PBS2	13.04 ± 0.09^b^	89.24 ± 3.78^c^	0.52 ± 0.15^b^	0.36 ± 0.04^a^	1.20 ± 0.12^cd^	29.94 ± 1.25^a^
SPS	13.65 ± 0.04^a^	94.45 ± 3.18^b^	0.41 ± 0.06^c^	0.25 ± 0.05^b^	1.10 ± 0.05^cd^	17.10 ± 0.67^e^
CF	11.21 ± 0.07^d^	96.53 ± 1.61^ab^	0.23 ± 0.02^de^	0.33 ± 0.03^ab^	1.05 ± 0.01^d^	17.53 ± 0.48^de^
CS	10.79 ± 0.02^e^	87.24 ± 1.78^c^	0.19 ± 0.04^e^	0.34 ± 0.05^ab^	1.47 ± 0.08^b^	18.96 ± 0.60^d^

*Note:* Values in the same column with different superscripts are significantly different at *p* < 0.05.

*All results in terms of starch components are based on a dry basis.

### 3.2. Morphology and Size Distributions of Starch Granules

SEM micrographs and particle size distributions are shown in Figure 1. As shown, *Pueraria* starch granules exhibited polygonal‐shaped granules with sharp edges, which are consistent with previous reports [18, 33]. In contrast, the other starch samples displayed a spherical shape. Notably, *Pueraria* starch granules differed from most tuber and root starches, with the exception of taro and elephant yam starches [34]. All tested starches showed a bimodal particle size distribution. The average particle size *D*[3, 4] of KS, PBS1, and PBS2 was 9.47, 8.91, and 8.96 *μ*m, respectively—smaller than that of SPS (18.96 *μ*m), CF (13.72 *μ*m), and CS (14.32 *μ*m)—which aligns with results reported in the literature [14, 35]. Liang et al. [2] documented that the average particle size of edible KS from Hunan, Guangxi, and Jiangxi in China is 8.8, 6.2, and 5.8 *μ*m, respectively, while that from Japan was 5.9 *μ*m. Several studies have indicated that starches with high amylose content possess distinct granule morphologies compared to normal amylose starches and typically exhibit smaller particle sizes [36], which is consistent with the compositional analysis results in this study. The differences in starch granule size and shape are attributed to biological origin, amyloplast biochemistry, and plant physiology [37]. Granule size, shape, and structure are known to influence starch functional properties such as swelling power, viscosity, thermal transition, and pasting behavior [14]. Specifically, the ability of starch granules to interact with water and hydrate at elevated temperatures is closely related to changes in swelling power, viscosity, and pasting properties, which vary with granule size [23]. Additionally, the hydrolysis rate of starch is proportional to the granule surface area: A larger surface area facilitates enzyme adsorption, resulting in more rapid hydrolysis of smaller granules compared to larger ones [38].

Figure 1(a) SEM micrographs, (b) particle size distributions, and (c) average particle size *D*[3, 4] of starches.

**Figure:**
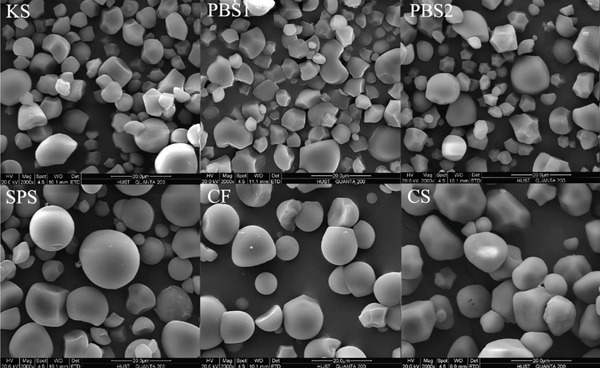
(a)

**Figure:**
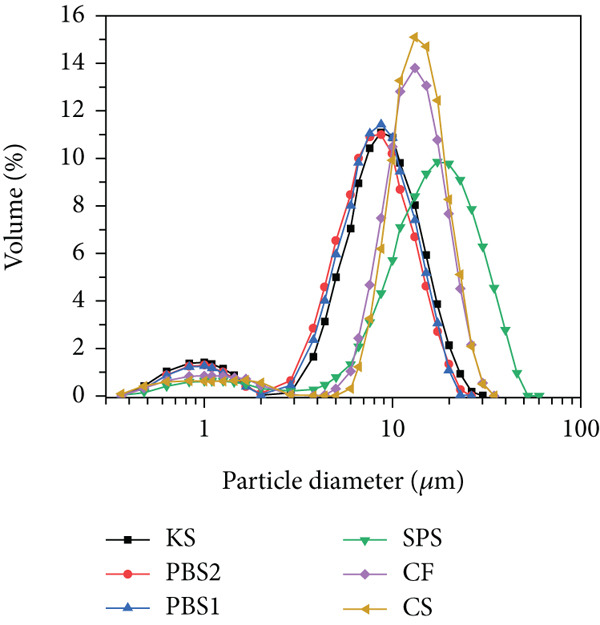
(b)

**Figure:**
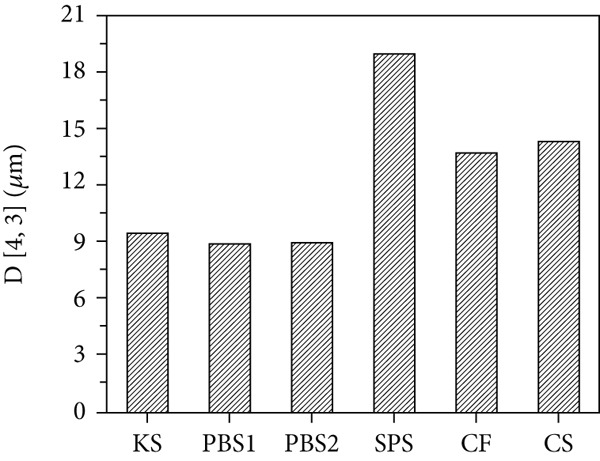
(c)

### 3.3. Crystalline Properties

**Figure 2:**
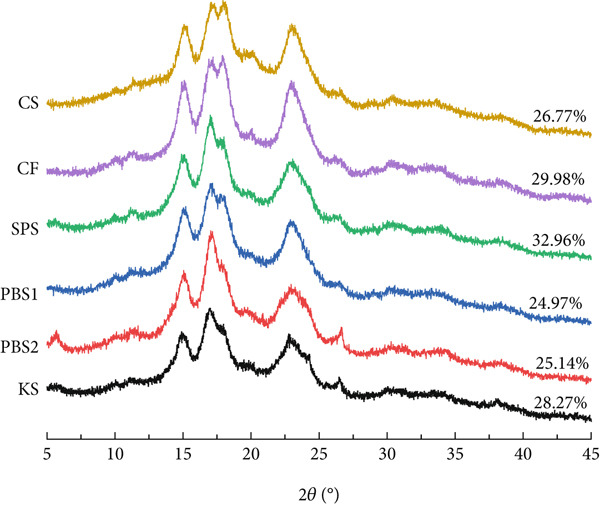
X‐ray diffraction patterns of starches.

The XRD patterns of starches are shown in Figure 2. KS, PBS1, PBS2, and SPS exhibited a C‐type crystallinity pattern, characterized by three strong diffraction peaks at 2*θ* 15.1°, 17.0°, and 23.1°, as well as two weak peaks at 2*θ* 17.9° and 26.6°. In contrast, CF and CS exhibited an A‐type crystallinity pattern, with diffraction peaks at 2*θ* 15.1°, 17.0°, 17.9°, and 23.1°. Most studies have reported that KS typically exhibits a C‐type crystallinity pattern [4, 37, 39], although A‐type and B‐type patterns have also been observed [14, 29, 40, 41]. Yoo et al. [42] further noted a C_A_‐type pattern for KS, which was more similar to the A‐type than to the B‐type. The crystallinity values of KS, PBS1, and PBS2 were 28.27%, 24.97%, and 25.14%, respectively, which were lower than those of CF (29.98%) and SPS (32.96%). Xia et al. [20] reported the crystallinity degrees of 32.4% for *Pueraria lobata* (Willd.) Ohwi and 43.5% for *Pueraria thomsonii* Benth. Discrepancies in the diffraction pattern and crystallinity may result from differences in genotype and growth conditions [18]. For soybean and SPSs, the polymorphic type can shift from B‐type or C‐type to A‐type with increasing temperature [43]. Crystallinity is affected by multiple factors, including amylopectin content and chain length, moisture content, double helices alignment, and crystallite size [14]. Additionally, starch–lipid complexes also affect the starch crystallinity [44]. Notably, a higher relative crystallinity of starch is associated with a lower hydrolysis index and estimation of glycemic index, with a significantly negative correlation observed between relative crystallinity and these two parameters [45]. Enthalpy is the characteristic related to the quantity of crystallinity within a starch molecule. The trend of enthalpy values was consistent with the relative crystallinity of starches. The variation in enthalpy values may result from differences in the intermolecular bonding forces between the double helices that constitute the crystalline structure of starch granules [14]. Furthermore, amylose influences starch water uptake, swelling, and gelatinization by regulating the packing of amylopectin into crystallites and the arrangement of the crystalline lamella within starch granules [23].

### 3.4. Pasting Properties

Pasting behavior supplies substantial details concerning the physiochemical properties of starches, offering a fundamental reference to facilitate the comprehension of how these starches can be potentially applied in processing. Pasting properties of starch samples are presented in Table 2. Pasting temperature of starches ranged from 70.37°C to 79.93°C, with the lowest value observed for CF and the highest for PBS1. The starches from *Pueraria thomsonii* Benth. (PBS1 and PBS2) exhibited significantly (*p* < 0.05) higher pasting temperature compared with kudzu and commercial starches, indicating greater resistance to the swelling and granule rupturing. This phenomenon was probably ascribed to higher crystallinity and double helical structures and more amylose content and more intermediate/longer amylose chains, which can hinder water molecules from penetrating starch granules, thereby increasing the pasting temperature [22]. However, short chains of amylopectin reduce the stability of the double helix in amylopectin molecules, consequently lowering the pasting temperature [36].

**Table 2:** Pasting properties of starches.

	**Pasting temperature (°C)**	**Viscosity (Pa·s)**				
		**Peak**	**Trough**	**Breakdown**	**Final**	**Setback**
KS	72.75 ± 0.09^d^	3.28 ± 0.04^b^	2.02 ± 0.07^a^	1.26 ± 0.04^d^	2.62 ± 0.06^a^	0.61 ± 0.05^b^
PBS1	79.93 ± 0.40^a^	3.48 ± 0.14^a^	1.97 ± 0.04^a^	1.51 ± 0.11^b^	2.55 ± 0.03^b^	0.57 ± 0.04^bc^
PBS2	78.07 ± 0.29^b^	3.15 ± 0.01^c^	1.76 ± 0.01^b^	1.38 ± 0.01^c^	2.31 ± 0.01^d^	0.54 ± 0.00^cd^
SPS	75.55 ± 0.00^c^	3.45 ± 0.03^a^	1.45 ± 0.01^c^	2.00 ± 0.02^a^	1.94 ± 0.02^e^	0.48 ± 0.02^e^
CF	70.37 ± 0.38^e^	3.17 ± 0.03^bc^	1.10 ± 0.01^d^	2.07 ± 0.01^a^	1.61 ± 0.03^f^	0.52 ± 0.02^de^
CS	75.83 ± 0.49^c^	2.22 ± 0.01^d^	1.72 ± 0.03^b^	0.50 ± 0.02^e^	2.41 ± 0.03^c^	0.69 ± 0.02^a^

*Note:* Values in the same column with different superscripts are significantly different at *p* < 0.05.

Peak viscosity, an index of starch granule resistance to swelling, was the highest viscosity during the heating. The peak viscosity of all starch samples ranged between 2.22 and 3.48 Pa·s, with CF exhibiting the lowest value and PBS1 the highest. Commonly, peak viscosity is regulated by the composition and structure of starches. Starch with high amylose content showed low peak viscosity due to restricted granule swelling [46]. However, some studies have reported that differences in the quantity of amylose were not sufficient to explain the variance in the peak viscosity of starches [32]. The trough viscosity of starches fluctuated between 1.10 and 2.02 Pa·s, the lowest value for CF and the highest for KS. Breakdown viscosity, the difference between peak and trough viscosity, reflects pasting stability, while setback viscosity, the increased viscosity resulting from the rearrangement of starch molecules, is symbolic of good cooking quality, as it implies cooked starches will neither retrograde nor stiffen during cooling. Compared with the *Pueraria* starches, SPS and CF showed lower trough viscosity and higher breakdown viscosity. Specifically, CF and SPS displayed the highest breakdown viscosity (2.00 and 2.07 Pa·s, respectively), whereas CS showed the lowest (0.50 Pa·s). Setback viscosity ranged from 0.48 to 0.69 Pa·s across all samples. Many factors influenced the pasting properties of starches, including amylose content, amylopectin branch chain length distribution, phosphate–monoester derivatives, lipids, and granule size [6, 47]. For instance, starches with a high proportion of A‐type polymorphs in C‐type starch, smaller granules, and long‐chain branched amylopectin tend to exhibit higher pasting temperatures [48]. However, it was difficult to explain the complete pasting profile with a single factor [49].

Pasting properties are critical considerations when selecting starches for use as gelling or thickening agents. Pasting properties are critical considerations when selecting starches for use as gelling or thickening agents. Starches such as SPS and CF, which possess relatively high peak viscosity, high breakdown viscosity, and low final viscosity, are unsuitable for these applications. In contrast, *Pueraria* starches are well suited as thickening or gelling agents in food processing. Compared with *Pueraria* starches, CS exhibited lower peak viscosity (2.22 Pa·s) and breakdown viscosity (0.50 Pa·s), while its final viscosity (2.41 Pa·s) was significantly higher than its peak viscosity. This indicates the poor stability of CS paste, which is consistent with previous reports [37].

### 3.5. Water and Oil Binding Capacities

Water binding capacity and oil binding capacity, which indicate the ability of starch to bind water or oil under external forces such as pressing, centrifugation, or heating, are presented in Figure 3a. Among the tested samples, PBS2 and KS exhibited the highest water binding capacities, at 99.87% and 98.64%, respectively, making them suitable for the processing of viscous foods. Water binding capacity is critical for starch swelling and gelatinization, which in turn contribute to the texture development of food products. Additionally, it influences the sensory characteristics (e.g., mouthfeel) and nutritional attributes (e.g., improving satiety and increasing food bulkiness) of foods. The water binding capacity of starch is affected by the amylose–amylopectin ratio, granule size, and granule structure. A loose association of amylopectin chains can enhance water binding capacity by providing more space for water molecules [32]. In contrast, the involvement of hydroxyl groups in forming hydrogen bonds and covalent bonds between starch chains reduces water binding capacity [50]. Regarding oil binding capacity, PBS1 showed the highest value (137.20%) with significant differences compared to other samples (*p* < 0.05), while SPS exhibited the lowest (76.76%). The oil binding capacities of KS and PBS2 were 108.20% and 108.64%, respectively. Starches with high oil binding capacity can improve mouthfeel, flavor retention, and palatability, as well as extend shelf life by slowing fat loss. Thus, they are suitable for scenarios requiring stable oil distribution, such as plant‐based meat products, emulsified sauces, baked and fried foods, fillings, and prepared dishes [44]. Oil binding capacity mainly depends on the content of amorphous protein and amylose, which bind lipids through their hydrophobic cavities [51].

Figure 3(a) Binding capacity of water and oil, (b, c) swelling power and solubility, and (d) freeze–thaw cycle of starches.

**Figure:**
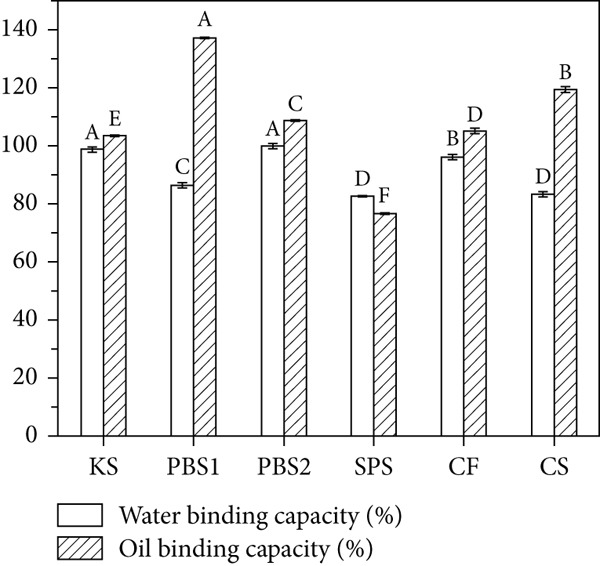
(a)

**Figure:**
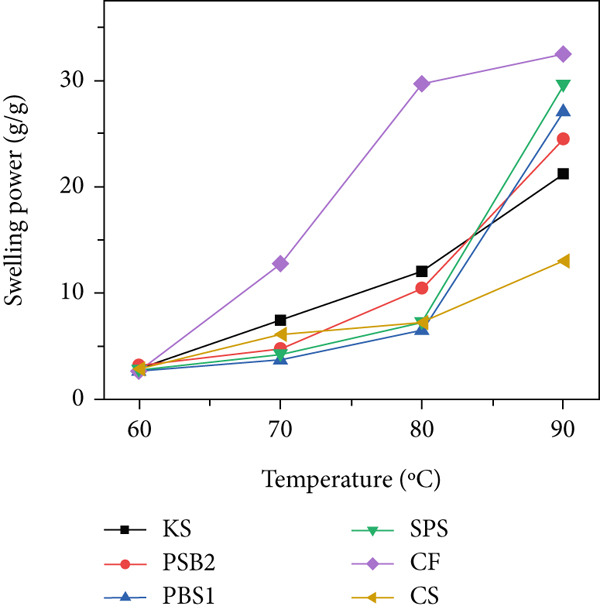
(b)

**Figure:**
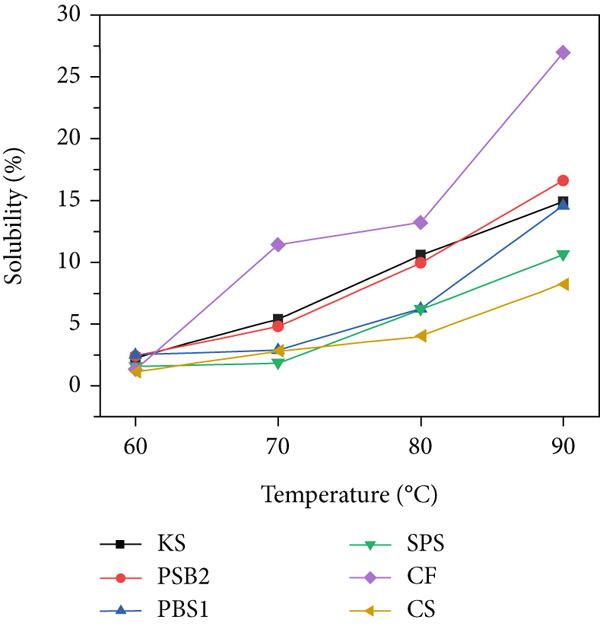
(c)

**Figure:**
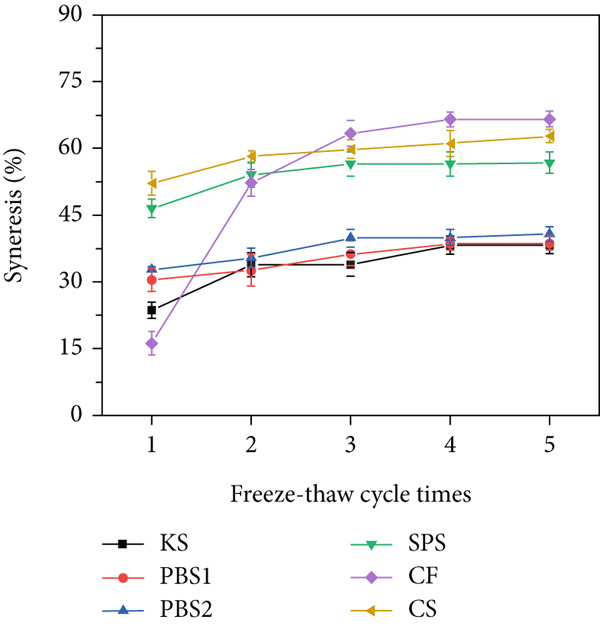
(d)

### 3.6. Swelling Power and Solubility

Swelling power and solubility, reflecting the interaction between the amylose molecules with water, are shown in Figure 3b,c. Both properties increased with rising temperature, consistent with previous findings on edible kudzu and KSs [14, 39]. As temperature increased, the chemical bonds within the granules loosened, leading to granule swelling and eventual collapse. Above 70°C, both swelling power and solubility increased significantly. At 90°C, the swelling power of KS, PBS1, and PBS2 was 21.21, 27.08, and 24.52 g/g, respectively, showing significant differences; their solubility was 14.90%, 14.60%, and 16.61%, respectively. CF exhibited high swelling power and solubility above 70°C, which was consistent with its pasting properties and indicated gelatinization onset at 70.37°C. Typically, starch swelling could be induced by the bond strength between molecules and by the molecular structure of amylopectin [52]. Starch solubility is primarily a result of amylose leaching, whereby amylose molecules segregate and disperse from the starch granules during the swelling process. The variations in swelling power and solubility among the tested starches during heating may stem from differences in amylose content, amylase–lipid complexes, the molecular structure of amylopectin, and the interaction between starch chains within the amorphous and crystalline regions of the granule [53]. Starch with low swelling power and solubility is suitable for application as edible coatings for fruits and vegetables.

### 3.7. Freeze–Thaw Stability

Freeze–thaw stability, evaluated by syneresis rate, reflected the water retention ability of the gel and was used as an indicator of the tendency of starch to retrograde. A lower syneresis rate denotes better freeze–thaw stability. The syneresis rates of all starch samples are presented in Figure 3d. CF exhibited the lowest initial syneresis rate (16.23%), but this value increased sharply to 52.25% after the second freeze–thaw cycle. After five freeze–thaw cycles, both CS and SPS showed relatively high syneresis rates. For KS, its syneresis rate (23.67%) was lower than that of PBS1 and PBS2 (30.44% and 32.76%, respectively) after the first freeze–thaw cycle. After five freeze–thaw cycles, the syneresis rates of KS, PBS1, and PBS2 were 38.34%, 38.59%, and 40.74%, respectively. These results indicate that *Pueraria* starches possess good freeze–thaw stability, making them desirable as stabilizers and thickeners in frozen food processing [23]. The high freeze–thaw stability of starch can ensure the textural quality of frozen food products throughout the production and distribution chain. Botanical source is a vital factor influencing the freeze–thaw stability of starch gels, which is attributed to differences in their structural characteristics. Specifically, amylose content and amylopectin branch chain length distribution also affect freeze–thaw stability [26]. Prediction models have illustrated that amylose content contributes positively to syneresis, while amylopectin branch chain length distribution contributes negatively. This phenomenon can be attributed to the long linear chains and high mobility of amylose molecules, as well as the high water holding capacity, highly branched structure, and short chains of amylopectin. The low freeze–thaw stability of starch could be improved through crosslinking and debranching modification [4, 41]. For instance, the syneresis rate of KS modified by debranching‐recrystallization treatment increased from 6.59% to 20.82% after five freeze–thaw cycles.

### 3.8. In Vitro Digestibility

The contents of RDS, SDS, and RS contents determined via in vitro enzymatic hydrolysis are presented in Table 3. All starches exhibited high RDS contents, ranging from 76.42% to 90.85%. Specifically, KS showed the highest RDS content (90.85%) accompanied by low SDS and RS contents (3.26% and 5.89%, respectively). In contrast, PBS1, PBS2, and SPS had relatively high SDS contents at 12.33%, 15.38%, and 15.78%, respectively. Among all samples, PBS1 possessed the highest RS content (11.26%), while KS and PBS2 had low RS contents (5.89% and 6.55%, respectively). Notably, CS exhibited the lowest RS content (3.05%) and a comparably high RDS content (90.62%). A previous study reported that starches with higher crystallinity tend to have lower digestion rates [22], which was inconsistent with the results obtained in this work. Generally speaking, starch granules with smaller sizes have larger specific surface areas, facilitating enzyme adsorption and thereby leading to rapid digestibility [6]. In addition, differences in starch digestibility may be attributed to the interplay of multiple factors, including starch source, granule size, amylose/amylopectin ratio, and phosphorus content [45]. Furthermore, the chain length distribution of amylopectin influences hydrolysis: chains with a DP of 8–12 and 16–26 show positive and negative correlations with hydrolysis, respectively [54]. Specifically, short amylopectin chains form short or weak double helices, resulting in inferior crystalline structures, whereas longer chains form extended helices that strengthen hydrogen bonding and hinder enzyme access.

**Table 3:** In vitro digestibility of starches.

	**RDS (%)**	**SDS (%)**	**RS (%)**
KS	90.85 ± 1.96^a^	3.26 ± 1.58^d^	5.89 ± 0.99^b^
PBS1	76.42 ± 1.98^c^	12.33 ± 2.27^b^	11.26 ± 0.99^a^
PBS2	78.08 ± 1.15^c^	15.38 ± 1.36^a^	6.55 ± 0.70^b^
SPS	78.72 ± 0.82^c^	15.78 ± 1.30^a^	5.50 ± 0.86^b^
CF	83.03 ± 0.51^b^	10.85 ± 1.02^b^	6.12 ± 1.24^b^
CS	90.62 ± 1.06^a^	6.33 ± 0.80^c^	3.05 ± 0.41^c^

*Note:* Values in the same column with different superscripts are significantly different at *p* < 0.05.

RS is a vital food component with multiple physiological benefits, including reducing the risk of colon cancer, constipation, and hemorrhoids; increasing fecal bulking; regulating blood glucose and cholesterol levels; and acting as a prebiotic to support the growth of beneficial probiotic bacteria [51]. Moreover, RS serves as a favorable substrate for butyrate production, and butyrate can alter microRNA (miRNA) levels in colorectal cancer cells, thereby mitigating the health risks associated with a high red meat diet [55]. Raw kudzu, which is characterized by high RS and low RDS contents, has been identified as suitable raw materials for developing SDS foods [38, 44, 56]. In contrast, gelatinized starches were nearly completely digested, with RS content below 10%. Controlling the accessibility of starch chains to digestive enzymes is critical for the production of low glycemic index foods based on *Pueraria* starches. Previous studies have demonstrated feasible strategies: Annealing treatment can modify the crystal structure and glass transition temperature of *Pueraria* starches, thereby increasing RS levels [57]; additionally, xanthan gum can coat the surface of starch granules, forming a physical barrier that impedes enzyme–starch interactions [58].

### 3.9. Correlation Analysis Among the Various Properties of Starches

**Figure 4:**
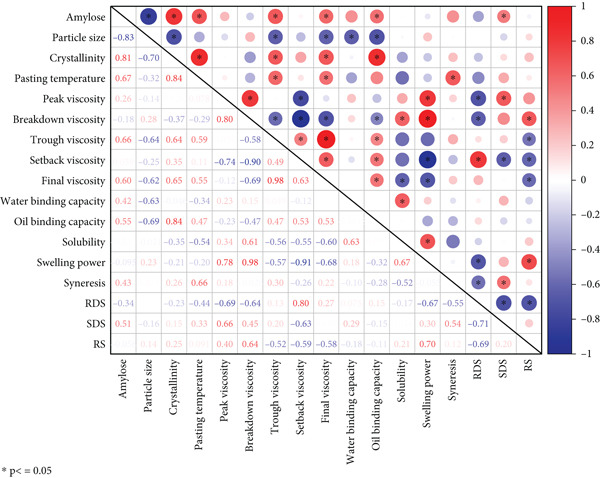
Pearson′s correlation among different properties of starch.

Pearson′s correlation analysis for the relationship among the various physicochemical properties of the starches is shown in Figure 4. Starch solubility exhibited a significant positive correlation with swelling power (*r* = 0.67, *p* ≤ 0.05) and water binding capacity (*r* = 0.63, *p* ≤ 0.05), which align with previous findings on SPSs [19]. In terms of amylose content, it showed a significant negative correlation with particle size (*r* = −0.83, *p* ≤ 0.05), while displaying significant positive correlations with crystallinity (*r* = 0.81, *p* ≤ 0.05), trough viscosity (*r* = 0.66, *p* ≤ 0.05), final viscosity (*r* = 0.60, *p* ≤ 0.05), oil binding capacity (*r* = 0.55, *p* ≤ 0.05), and SDS content (*r* = 0.51, *p* ≤ 0.05). No obvious correlation was observed between amylose content and peak viscosity in the present data. Some studies showed that there was an insignificant correlation between amylose content and average particle size and pasting properties [59]. However, the ratio of relative molar distribution of amylopectin unit chains with DP 6–12 to that of DP 6–24 positively correlated with swelling power and breakdown viscosity but negatively with pasting temperature, peak time, peaking viscosity, hot paste viscosity, final viscosity, and setback viscosity. Particle size exhibited a significant negative correlation with crystallinity (*r* = −0.70, *p* ≤ 0.05), as well as with parameters including water binding capacity (*r* = −0.63, *p* ≤ 0.05) and oil binding capacity (*r* = −0.69, *p* ≤ 0.05). For starch crystallinity, it was significantly and positively correlated with oil binding capacity (*r* = 0.84, *p* ≤ 0.05). The particle size of starch was important in determining the pasting and other properties. Previous studies showed that the particle size of the starch significantly correlated with peak viscosity, breakdown viscosity, and final viscosity [60]. Syneresis showed positive correlations with pasting temperature (*r* = 0.66, *p* ≤ 0.05) in the present study. Srichuwong et al. [26] pointed out that the syneresis of starch gels largely depended on their differences in structural features. The optimal multiple linear regression models showed a positive unit contribution of apparent amylose content and a negative unit contribution of APC ratio (proportional ratio of amylopectin branch chains DP 6–12 to those of DP 6–24) to syneresis.

Pasting temperature showed positive correlations with trough viscosity (*r* = 0.59, *p* ≤ 0.05), final viscosity (*r* = 0.55, *p* ≤ 0.05), amylose content (*r* = 0.67, *p* ≤ 0.05), and crystallinity (*r* = 0.84, *p* ≤ 0.05). Peak viscosity exhibited significant positive correlations with breakdown viscosity (*r* = 0.80, *p* ≤ 0.05), swelling power (*r* = 0.78, *p* ≤ 0.05), and SDS content (*r* = 0.66, *p* ≤ 0.05) but a significant negative correlation with setback viscosity (*r* = −0.74, *p* ≤ 0.05) and RDS content (*r* = −0.69, *p* ≤ 0.05). Breakdown viscosity showed significant negative correlations with trough viscosity (*r* = −0.58, *p* ≤ 0.05), setback viscosity (*r* = −0.90, *p* ≤ 0.05), final viscosity (*r* = −0.69, *p* ≤ 0.05), oil binding capacity (*r* = −0.47, *p* ≤ 0.05), and RDS content (*r* = −0.64, *p* ≤ 0.05), alongside significant positive correlations with solubility (*r* = 0.61, *p* ≤ 0.05) and swelling power (*r* = 0.98, *p* ≤ 0.05). Finally, setback viscosity displayed significant positive correlations with final viscosity (*r* = 0.63, *p* ≤ 0.05), oil binding capacity (*r* = 0.53, *p* ≤ 0.05), and RDS content (*r* = 0.80, *p* ≤ 0.05) while showing significant negative correlations with solubility (*r* = −0.55, *p* ≤ 0.05), swelling power (*r* = −0.91, *p* ≤ 0.05), SDS content (*r* = −0.63, *p* ≤ 0.05), and RS content (*r* = −0.59, *p* ≤ 0.05). Using simple linear regression analysis, insignificant correlations were found, but the multiple regression analysis revealed the extent of amylose content, average granule size, and pasting properties [59].

## 4. Conclusions

*Pueraria* starches exhibited higher amylose contents (22.92% for KS, 27.75% for PBS1, and 29.94% for PBS2) and polygonal granules with sharp edges, accompanied by smaller average particle sizes (8.91–9.47 *μ*m). All tested starches presented a bimodal particle size distribution. KS, PBS1, PBS2, and SPS displayed a C‐type crystallinity pattern, whereas CF and CS exhibited an A‐type pattern; the crystallinity of *Pueraria* starches (24.97%–28.27%) was lower than that of CF (29.98%) and SPS (32.96%). Among the samples, *Pueraria thomsonii* Benth. starches had significantly higher pasting temperatures (*p* < 0.05). In addition, *Pueraria* starches showed moderate peak viscosity (2.83–3.48 Pa·s), low breakdown viscosity, and stable final viscosity—properties that make them superior to commercial starches for gelling/thickening applications. *Pueraria* starches also had lower syneresis rates (38.34%–40.74% after five freeze–thaw cycles) than commercial starches, indicating excellent stability for frozen food processing. Among the *Pueraria* starches, PBS1 exhibited the highest RS content (11.26%). Correlation analysis revealed that amylose content was positively correlated with crystallinity (*r* = 0.81, *p* ≤ 0.05), oil binding capacity (*r* = 0.55, *p* ≤ 0.05), and SDS content (*r* = 0.51, *p* ≤ 0.05) but negatively correlated with particle size (*r* = −0.83, *p* ≤ 0.05). Meanwhile, breakdown viscosity was strongly positively correlated with swelling power (*r* = 0.98, *p* ≤ 0.05) and negatively correlated with setback viscosity (*r* = −0.90, *p* ≤ 0.05), which highlights the interdependence of starch properties.

This research establishes a theoretical foundation for developing *Pueraria* starch resources in both food and nonfood industry applications. For example, in the field of frozen foods, their low syneresis rate enables them to serve as ideal stabilizers and thickeners for products such as ice cream and frozen soups, effectively preventing the textural deterioration that often occurs during repeated freeze–thaw cycles. However, this study had limitations in structural analysis depth: Although XRD and SEM provided basic insights into the crystalline structure and granule morphology of the starches, advanced analytical techniques (e.g., small‐angle x‐ray scattering, SAXS; nuclear magnetic resonance, NMR) were not used to analyze fine‐scale structural features, such as amylopectin branch chain length distribution and double‐helix density.

## Conflicts of Interest

The authors declare no conflicts of interest.

## Author Contributions

Jianbin Shi and Haofeng Zou contributed equally to this work.

## Funding

This study was funded by the Hubei Province Rural Revitalization Science and Technology Support Project (2022BBA088).

## Data Availability

Data will be made available on request.
